# Comprehensive Analysis of Aberrant m6A RNA Modifications Identifies Prognostic Biomarkers in Non-Small Cell Lung Cancer

**DOI:** 10.7150/ijms.119651

**Published:** 2025-10-24

**Authors:** Yifei Li, Peng Jiao, Donghang Li, Yi Tian, Hexin Li, Gaoyuan Sun, Xiaonan Wu, Xin Nie, Xu Li, Siyuan Xu, Xiaokun Tang, Lili Zhang, Li Wan, Lanxin Zhang, Jiahui Cai, Min Tang, Lin Li

**Affiliations:** 1 Clinical Biobank, Beijing Hospital, National Center of Gerontology, Institute of Geriatric Medicine, Chinese Academy of Medical Sciences, Beijing, 100730, People's Republic of China.; 2 Department of Thoracic Surgery, Beijing Hospital, National Center of Gerontology, Institute of Geriatric Medicine, Chinese Academy of Medical Sciences, Beijing, People's Republic of China.; 3 Department of Medical Oncology, Beijing Hospital, National Center of Gerontology, Institute of Geriatric Medicine, Chinese Academy of Medical Sciences, Beijing, People's Republic of China.

**Keywords:** Non-small cell lung cancer, N^6^-methyladenosine, Long-read sequencing, Prognosis, Biomarker

## Abstract

**Background:** Dysregulation of N6-methyladenosine (m6A) RNA modification plays a critical role in the development and progression of non-small cell lung cancer (NSCLC).

**Methods:** To explore the m6A modification landscape in NSCLC, we utilized direct RNA nanopore sequencing (dRNA-seq) to compare m6A patterns between NSCLC and adjacent normal tissues.

**Results:** Our analysis revealed distinct m6A modification differences, with tumor tissues showing reduced m6A density compared to normal tissues. Aberrantly modified genes, such as *SOX2* and *TOP2A*, exhibited hypomethylated m6A modifications and were upregulated in NSCLC tissues. We identified 14,419 differentially methylated m6A sites, with 49.5% hypermethylated and 50.5% hypomethylated. Functional enrichment analysis showed that hypermethylated genes were involved in DNA replication and transcription regulation, while hypomethylated genes were linked to cell migration and MAPK signaling. The expression patterns of m6A regulators, including *METTL3*, *METTL16*, *CBLL1*, *FTO*, *ALKBH5*, and *ELAVL1*, were consistent across NSCLC subtypes. Furthermore, correlation with clinical data from the TCGA database revealed that m6A-associated DEGs, such as *HMGA1*, *ERO1A*, *LRFN4*, *SNTN*, *SLC2A1*, *DNASE2B*, and *VSIG2*, were prognostically significant in NSCLC.

**Conclusions:** This study underscores the pivotal role of m6A modifications in NSCLC and highlights the potential of dRNA-seq for identifying RNA epigenetic changes that may serve as novel therapeutic targets.

## Introduction

Lung cancer is the leading cause of cancer-related deaths worldwide, with non-small cell lung cancer (NSCLC) representing 80-85% of cases^1^. The major NSCLC subtypes include adenocarcinoma (LUAD), squamous cell carcinoma (LUSC), and large cell carcinoma (LCC). Advances in molecular pathology have deepened our understanding of NSCLC's pathophysiology and heterogeneity, leading to significant progress in treatment, particularly with targeted therapies and immunotherapies that have improved outcomes for many patients^2^-^5^. However, challenges such as tumor heterogeneity, drug resistance, and a lack of validated biomarkers continue to limit the efficacy and accessibility of these therapies^6^-^8^. To address these issues, a better understanding of molecular biomarkers, along with the development of combination therapies, is essential for providing effective and personalized treatment^9^, particularly in resource-limited settings and vulnerable populations^10^,^11^.

N6-methyladenosine (m6A) is the most prevalent internal modification of RNA, playing a crucial role in regulating RNA metabolism, including splicing, stability, translation, and degradation^12^,^13^. m6A modifications are dynamically controlled by "writers" (methyltransferases), "erasers" (demethylases), and "readers" (binding proteins), which together orchestrate RNA regulation^14^. Dysregulation of m6A has been implicated in the development and progression of various cancers, including lung cancer, affecting tumor growth, metastasis, and drug resistance^15^-^17^. For instance, m6A modifications can regulate key oncogenes such as *MYC*, and alterations in m6A regulators like *METTL3* are linked to poor prognosis in lung cancer patients^18^. However, while the role of m6A in cancer is well studied, its specific contributions to non-small cell lung cancer (NSCLC), particularly in the tumor microenvironment and adjacent normal tissues, remain poorly understood^19^. Previous studies have primarily focused on individual m6A regulators or limited datasets, highlighting the need for comprehensive analyses integrating multi-dimensional RNA sequencing techniques, such as MeRIP-seq and RNA-seq, to fully elucidate the regulatory landscape of m6A in NSCLC^20^,^21^.

Direct RNA nanopore sequencing (dRNA-seq) using Nanopore technology offers significant advantages for m6A research compared to conventional RNA sequencing methods^22^. Unlike traditional techniques that rely on reverse transcription to generate cDNA, nanopore sequencing directly analyzes RNA molecules, preserving native modifications such as m6A. This enables a more accurate profile of RNA modifications across the transcriptome^23^. Additionally, nanopore sequencing generates long reads, allowing for the detection of m6A modifications within full-length transcripts and improving the resolution of m6A sites in complex RNA structures^24^. Its single-nucleotide resolution capability makes it particularly valuable for cancer research, providing insights into gene expression regulation, RNA metabolism in tumors, and the development of novel therapeutic strategies^25^,^26^.

In this study, we employed direct RNA nanopore sequencing to investigate aberrantly m6A-modified genes and differentially expressed genes (DEGs) between NSCLC and its adjacent normal tissues. We identified 223 m6A-associated DEGs. Notably, the m6A modification levels in these genes were significantly correlated with their transcriptional activity, highlighting the impact of m6A modification on gene expression. Nine m6A regulator-associated dysregulated genes were further identified, all of which were linked to prognosis based on analysis of The Cancer Genome Atlas (TCGA) clinical data. Our findings demonstrate the potential of nanopore sequencing for integrated analysis of m6A modifications and RNA expression, providing a valuable approach for identifying potential therapeutic targets.

## Methods

### Sample Collection and Long-read Sequencing

Tissue samples were surgically collected five treatment-naive patients diagnosed with NSCLC at Beijing hospital. Total RNA was extracted using the TRIzol™ Reagent (Thermo Fisher Scientific, USA) following the manufacturer's protocol and purified with the Dynabeads™ mRNA Purification Kit (Invitrogen, USA). Direct RNA sequencing was conducted using the SQK-RNA004 chemistry kit (Oxford Nanopore Technologies, UK). RNA quantity was assessed via Nanodrop 2000 and Qubit RNA/dsDNA HS Assay Kits (Thermo Fisher Scientific, USA), and RNA integrity was evaluated using the Agilent 5200 and DNF-471 RNA Kit (Agilent, USA). RNA libraries were prepared and sequenced on the Nanopore GridION platform with R10.4.1 flow cells (Oxford Nanopore Technologies). Basecalling was performed using Guppy software (version 6.0.1) with the SUP model. Raw sequencing data have been deposited in the Genome Sequence Archive for Human (GSA-Human; https://ngdc.cncb.ac.cn/gsa-human) under accession number HRA010114 and are publicly accessible.​​ This study was approved by the Ethics Committee of Beijing Hospital, and informed consent was obtained from all participants.

### Analysis of Whole m6A Sites

The assembled sequences were aligned to the human genome (GRCh38) using Minimap2 (version 2.22)^27^. m6A modification levels were determined with m6Anet (version 2.1.0) at single-nucleotide resolution^28^. The m6A ratio at each site was calculated by dividing the number of modified reads by the total reads, using a threshold of 0.9 to define true m6A sites. A sliding window of 1M was applied to visualize the distribution of m6A sites across the genome, with data visualized using the RIdeogram R package^29^. Peak distributions in functional regions, such as 5' UTR, CDS, and 3' UTR, were analyzed with the Guitar R package.

### Analysis of m6A Modifications

To compare m6A modification patterns between tumor and adjacent normal tissues, differential m6A modification rates were calculated using Xpore (version 2.0)^30^. Differentially modified regions were identified based on an absolute modification rate change > 0.5 and a *P* < 0.001. Statistical analyses of m6A site density between tumor and normal tissues was performed using the Mann-Whitney U test.

### Analysis of Differentially Expressed Genes (DEGs)

Nanopore reads were aligned to the human genome (GRCh38), and gene expression levels were quantified using NanoCount (version 1.0.0) to generate a normalized gene expression matrix 31. Genes with fewer than 5 reads in at least 3 samples were excluded. DEGs were identified with false discovery rate (FDR) < 0.05 and |log_2_(fold change)| > 1.5. Principal component analysis (PCA) was conducted using the R package 'models,' and transcript expression levels were quantified as transcripts per kilobase per million mapped reads (TPM) for cross-sample comparisons. Functional enrichment analysis of downregulated m6A-modified genes was performed using Gene Ontology (GO) terms (biological process, molecular function, and cellular component) and Kyoto Encyclopedia of Genes and Genomes (KEGG) pathway analysis, implemented on the DAVID bioinformatics platform.

**Statistical Analysis**

Clinical and transcriptomic data were obtained from The Cancer Genome Atlas (TCGA) lung squamous cell carcinoma (LUSC, n=622) and lung adenocarcinoma (LUAD, n=645) cohorts. To mitigate confounding from perioperative mortality, patients with overall survival (OS) time ≤30 days were excluded (n=330). Gene expression thresholds for prognostic stratification were determined using the ​maximally selected rank statistics algorithm​ implemented in the surv_cutpoint function (survminer), which optimizes cutpoints to maximize survival discrimination while enforcing a minimum subgroup proportion of 10%. Kaplan-Meier survival curves were generated via the survfit function (survival) with log-rank testing for between-group differences. Survival data visualization utilized ggsurvplot (survminer). All analyses were conducted in R (v4.2.1).

## Results

### Analysis of m6A Modification Landscape in NSCLC

**Figure 1:**
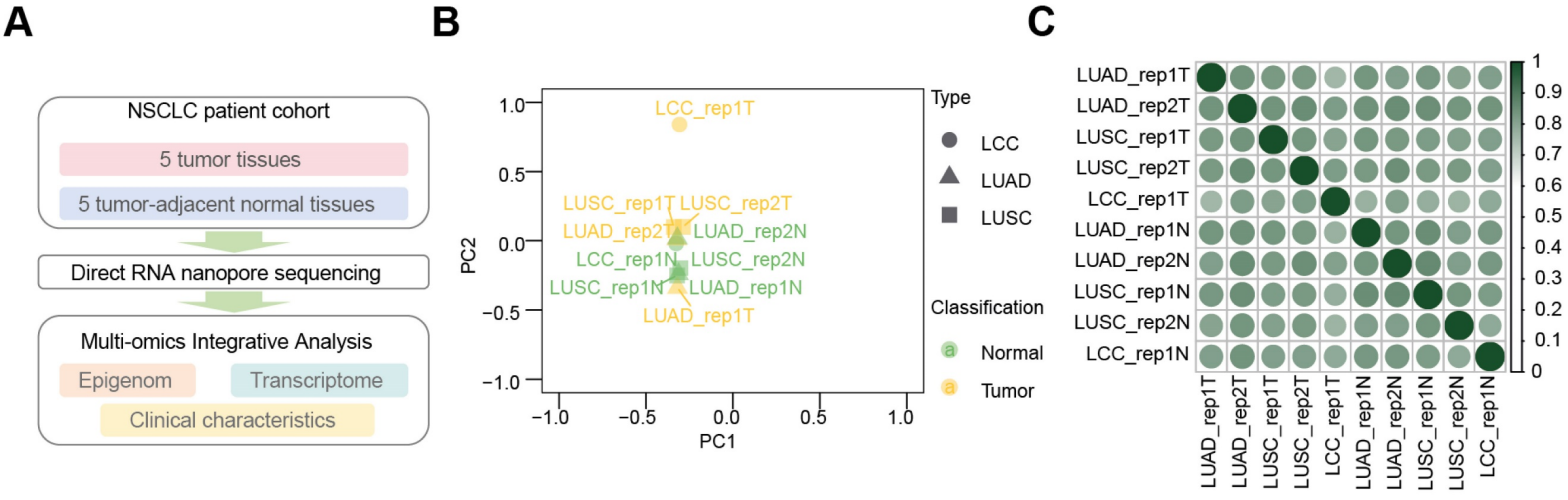
** Comprehensive analysis of dRNA-seq data from NSCLC tissues and their adjacent normal tissues. (A)** Overview of m6A multi-omics profiling integration in NSCLC issues and adjacent normal tissues. **(B)** Principal component analysis (PCA) showing tumors (yellow) and adjacent normal tissues (green) across LUAD (triangles), LUSC (square), and LCC (dots) in dRNA-seq.** (C)** Heatmap of Pearson correlation coefficients across ten samples in dRNA-seq. Color scale from white to green represents correlation strength (weak to strong).

Using the dRNA-seq approach, we profiled m6A modification landscapes in five primary NSCLC tissues and their adjacent normal tissues (Figure 1A). Principal component analysis (PCA) of LUAD, LUSC, and LCC samples revealed that tumor and normal samples clustered separately, with tumor samples from different subtypes showing close association, as did the normal samples (Figure 1B). Pearson correlation analysis (Figure 1C) indicated high correlation among tumor samples (r > 0.6) and even higher correlation among normal samples (r > 0.9). Notably, the correlation coefficients for LUAD (r > 0.77), LCC (r > 0.63), and LUSC (r > 0.40) tumor samples compared to adjacent normal tissues suggest a potential link to the transformation of normal lung tissue into NSCLC.

### m6A Modifications and Their Correlations with Gene Expression in NSCLC

**Figure 3:**
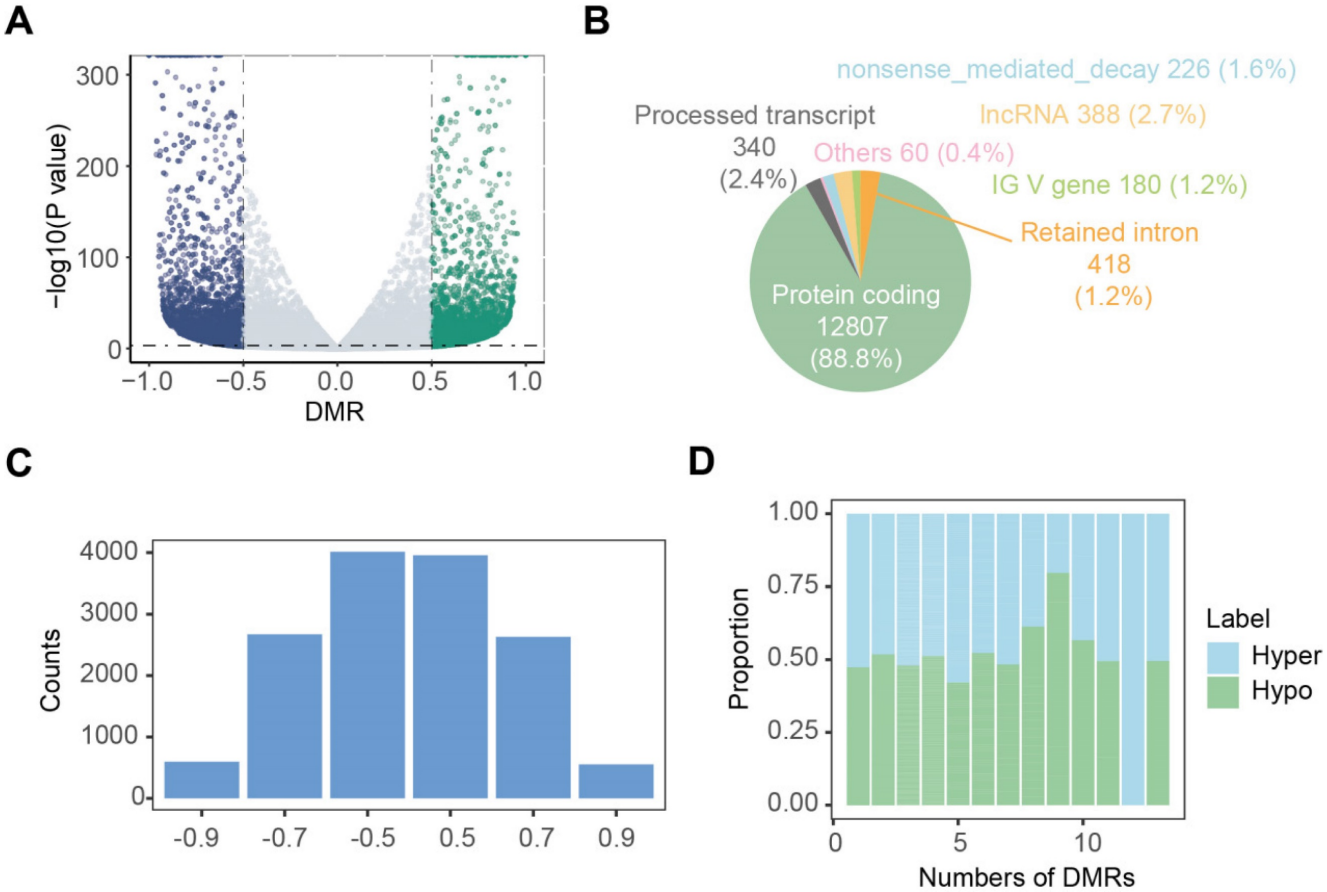
** Landscape of aberrant m6A modifications in NSCLC tissues. (A)** Volcano plot showing hyper- (green) and hypo- (blue) m6A modification-related genes in NSCLC tissues compared to normal tissues. **(B)** Types of genes with aberrant m6A modifications. **(C)** Distribution of genes based on m6A ratio. **(D)** Proportion of hyper- and hypo- m6A sites across different numbers of DMRs.

Nanopore dRNA-seq data were used to analyze m6A modifications and RNA transcription. By calculating the differential modification rate (DMR), which represents the difference between the modification rates in the tumor and control groups, using A-centered k-aggregates with xPore (NNANN), we identified a total of 14,419 methylated sites with high confidence (|DMR| > 0.5, *P* < 0.001), of which 49.5% (7138) were hypermethylated m6A sites; and 50.5% (7281) were hypomethylated sites (Figure 3A; Table S1). Next, we compared the m6A modification profiles between tumor and tumor-adjacent normal tissues, focusing on genes with aberrant m6A modification patterns. Figure 3B shows that 88.8% of these aberrantly m6A-modified genes belonged to protein-coding genes, suggesting their involvement in RNA transcription and translation. In Figure 3C, we analyzed the distribution of m6A modification changes and observed more hyper-methylated genes in tumor-adjacent normal tissues (n = 7,281) than in tumor tissues (n = 7,138). Additionally, for genes with varying numbers of m6A sites, the majority (96.7%) exhibited fewer than 5 m6A sites (Figure 3D). Furthermore, most genes (54.1%) with more than 5 m6A sites showed upregulation of m6A in the tumor tissue.

**Figure 4:**
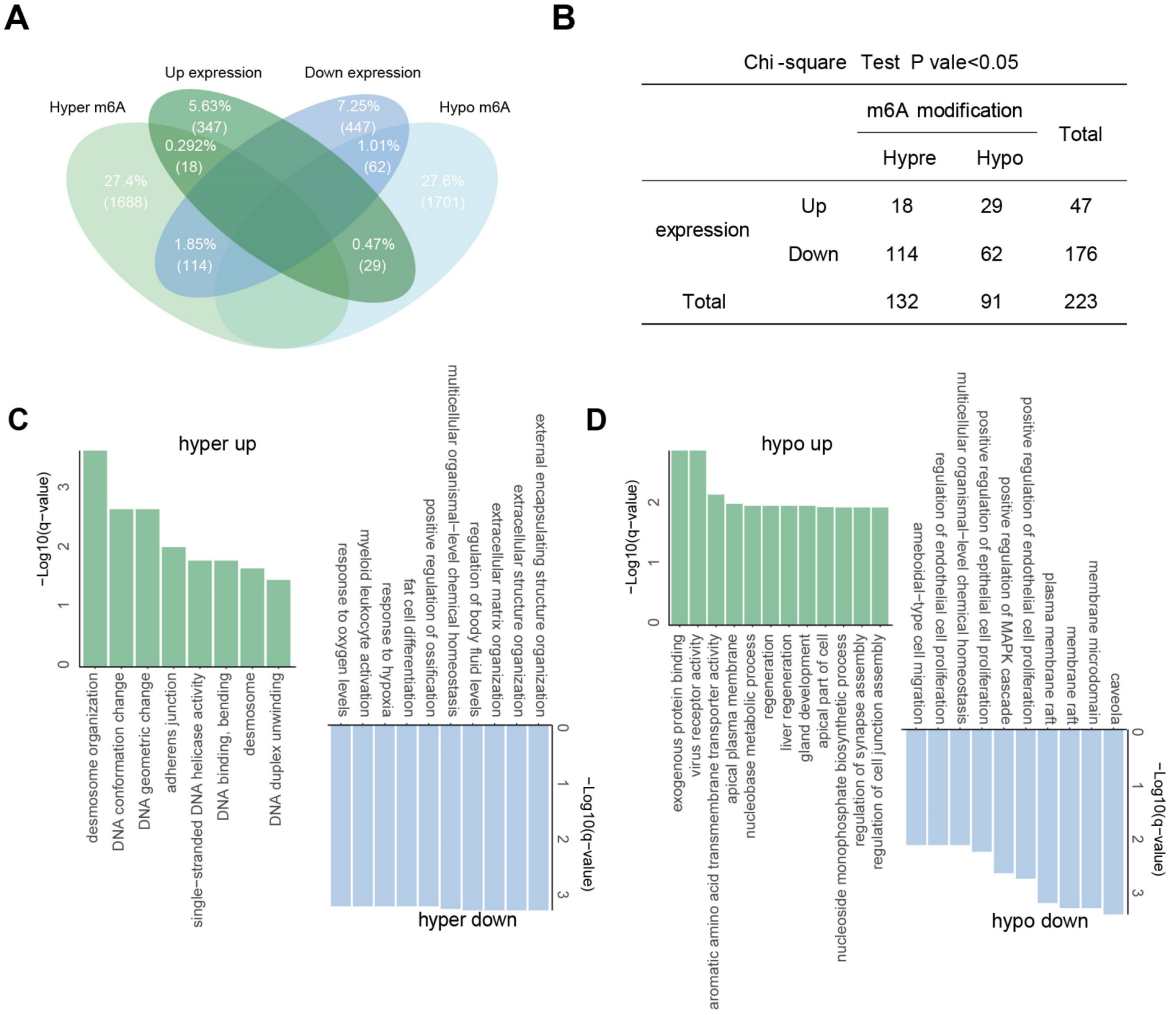
** Association between m6A modifications and gene expression. (A)** Intersection of aberrantly m6A-modified genes and differentially expressed genes. **(B)** Fisher's exact test showing the association between aberrant m6A modification and differential gene expression. **(C-D)** GO enrichment for genes with hyper- **(C)** and hypo-** (D)** m6A modifications.

To identify genes potentially regulated by m6A modifications, we performed a co-differential analysis integrating RNA-seq and dRNA nanopore sequencing data. Genes meeting both of the following criteria were defined as “m6A-associated DEGs”: significant differential expression (|log_2_(fold change)| > 1.5, *P* < 0.05) in RNA-seq and significant differential m6A modification (DMR > 0.5, P < 0.001) in nanopore dRNA-seq. A total of 223 significant co-differential genes were filtered, which were regarded as “m6A-associated DEGs”. The largest group consisted of 114 genes with significant hyper-methylation and down-regulated gene expressions. Following this, 62 genes showed significant hypo-methylation with down-regulated expression, 29 genes had significant hypo-methylation with up-regulated expression, and 18 genes had significant hyper-methylation with up-regulated expression (Figure 4A). To investigate whether m6A modification can influence gene expression, a chi-square test was conducted on these m6A-associated DEGs, which showed that the levels of m6A in m6A-associated DEGs were highly correlated with their transcription (Fisher's exact test, *P* = 0.0014) (Figure 4B). Notably, genes such as *SOX2* and *TOP2A* displayed hypomethylated m6A modifications coupled with transcriptional upregulation in tumors, while *SFTPC* and *GPM6A* showed hypomethylation with downregulation. *GJB5*, *IGHV1-24*, and *S100A2* were the significantly hypermethylated-upregulated genes, and *MCEMP1*, *FABP4*, and *DES* were the significantly hyper-down regulated genes (Table S2).

### Functional enrichment analysis of m6A-associated DEGs in NSCLC

We then performed GO enrichment analysis for these m6A-associated DEGs to gain insights into the significance of m6A modification changes in NSCLC (Figure 4C and D). The common GO biological process categories of hypomethylated-upregulated and hypermethylated-upregulated genes were enriched in DNA duplex unwinding, DNA geometric change, DNA conformation change, and desmosome organization. The common GO biological process categories of hypo-down and hyper-down related genes were enriched in positive regulation of MAPK cascade, ameboidal-type cell migration, and epithelial cell proliferation.

### Expression Patterns of m6A Regulators in NSCLC

**Figure 5:**
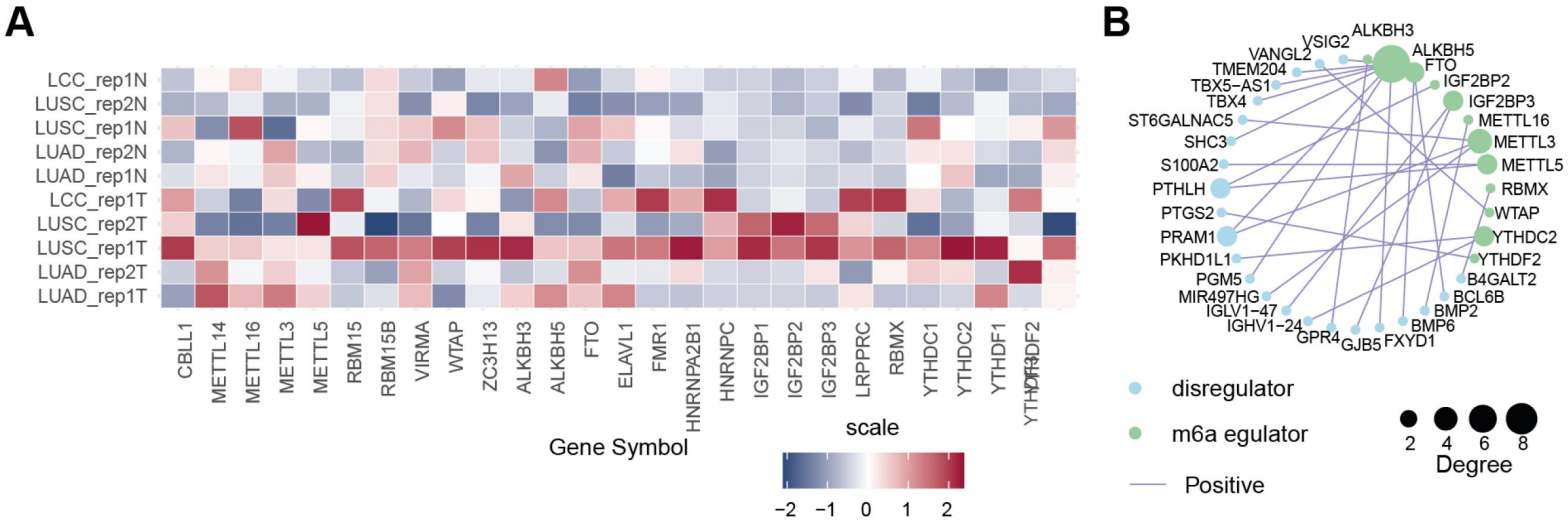
** Expression patterns and regulatory networks of m6A regulators in NSCLC.** (**A**) Heatmap of expression profiles of m6A writers, erasers, and readers in tumors and tumor-adjacent normal tissues, based on dRNA-seq data. The color scale represents log_2_(TPM + 1) values for each m6A regulator. **(B)** Interaction map showing the correlation (r > 0.95) between 27 m6A regulators and 23 m6A-associated DEGs in NSCLC, with positive correlations in purple lines. Dot size represents the degree of correlation.

According to a literature review, 27 m6A regulator genes were detectable in our dRNA nanopore data, consisting of 10 m6A writers (*CBLL1*, *METTL3*, *METTL5*, *METTL14*, *METTL16*, *RBM15*, *RBM15B*, *VIRMA*, *WTAP*, and *ZC3H13*), 3 m6A erasers (*ALKBH3*, *ALKBH5* and *FTO*), and 14 m6A readers (*ELAVL1*, *FMR1*, *HNRNPA2B1*, *HNRNPC*, *IGF2BP1*, *IGF2BP2*, *IGF2BP3*, *LRPPRC*, *RBMX*, *YTHDC1*, *YTHDC2*, *YTHDF1*, *YTHDF2*, and *YTHDF3*) (Figure 5A). We further combined the average of TPM + 1 from five pairs of normal samples and tumor samples in dRNA nanopore and created a heatmap after converting to log_2_(TPM + 1) (Figure 5A). The heatmap shows that these 27 m6A regulator genes exhibited diverse expression patterns, whether categorized as m6A writers, erasers, or readers, or analyzed in terms of their expression patterns in dRNA nanopore sequencing, indicating that the m6A regulatory mechanism in tumor samples is highly complex and heterogeneous. However, it is still evident that some m6A regulator genes had similar expression patterns in both dRNA nanopore sequencing data, and in terms of expression levels. Examples included *METTL3*, *METTL16*, and *CBLL1* among m6A writers, *FTO* and *ALKBH5* among m6A erasers, and *ELAVL1* among m6A readers. The above analyses showed that the genetic and expressional variations in m6A regulators were highly heterogeneous between NSCLC and adjacent tissues, suggesting a crucial role for the imbalance of m6A regulator expression in the development and progression of NSCLC. Moreover, the function of genes is not isolated, in that it has been shown that collaboration among m6A regulators exists in the context of cancer. Thus, the correlation of m6A-associated DEGs discovered in this study and m6A regulators were explored. We identified a notable co-expression trend between m6A-associated DEGs and 88.9% (n = 24) of the known m6A regulators (Table S3), with Pearson correlation coefficients (r) exceeding 0.95 were shown as Figure 5B, suggesting an essential role of m6A regulators (*ALKBH5*, *RBMX*, *METTL5*, *WTAP*, *YTHDF2*, *ALKBH3*, *METTL3*, *METTL16*, *FTO*, *YTHDC2*, *IGF2BP2*, and *IGF2BP3*) in modulating the expressions of m6A-associated DEGs. Taken together, these findings highlighted the important cross-talk roles among m6A regulators in the formation of distinct m6A modification patterns (Table S3).

### Prognostic significance of m6A-associated DEGs in NSCLC

**Figure 6:**
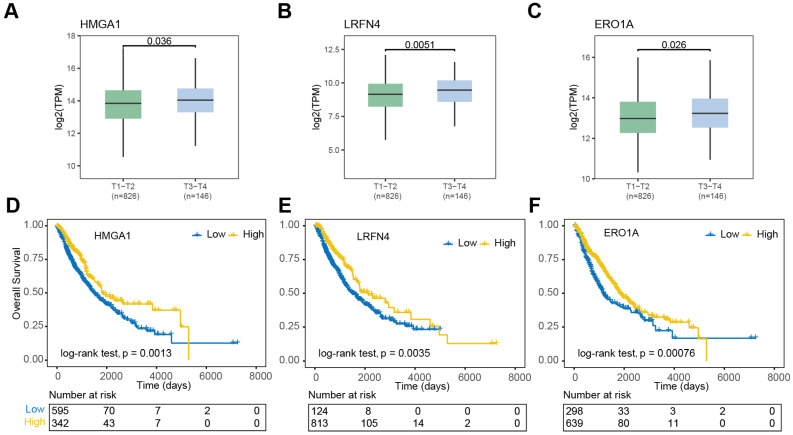
** Clinical and prognostic significance of m6A-associated DEGs in NSCLC. (A-C)** Expression levels of *HMGA1*, *LRFN4*, and *ERO1A* in early (T1-T2) vs. advanced (T3-T4) NSCLC. Bars represent mean ± SEM. **(D-F)** Kaplan-Meier survival curves for NSCLC patients stratified by high (red) vs. low (blue) expression of *HMGA1*, *LRFN4*, and *ERO1A*. Log-rank P values indicate significant prognostic associations.

To explore the potential prognostic significance of m6A regulator-associated m6A-DEGs (|log_2_(fold change)| > 1.5, *P* < 0.05 & DMR > 0.5, *P* < 0.001) in NSCLC, we analyzed data from the TCGA database. Notably, differential expression patterns were observed in relation to the pathologic T stage of the disease. In contrast, *HMGA1*,* LRFN4*, *SLC2A1*, and *ERO1A* exhibited significantly higher expression levels in NSCLC patients in pathologic T_3_-T_4_ stages compared to those in T_1_-T_2_ pathologic stages (Figure 6A-C). Conversely, *DNASE2B*, *SNTN*, and *VSIG2* exhibited significantly higher expression levels in the T_1_-T_2_ group compared to in the T_3_-T_4_ group (Figure S2A). Furthermore, nine m6A regulator-associated m6A-DEGs showed significant correlations with patient prognosis. The OS curves presented in Figure 6D-F indicate that high expressions levels of *HMGA1*,* LRFN4*, *PKP3*, *DNASE2B*, *SNTN*, *VSIG2*, and *ERO1A* were associated with an impaired prognosis (log-rank p < 0.05) for NSCLC patients. In contrast, higher expression levels of *SLC2A1* and *VGLL3* were linked to favorable prognosis (log-rank p < 0.05) (Figure S3A).

## Discussion

This study offers new insights into the role of m6A RNA modification in NSCLC by analyzing differential m6A modifications between tumor and adjacent normal tissues. Using direct RNA nanopore sequencing, we observed decreased m6A levels in tumor tissues compared to normal tissues, suggesting that m6A modification may have a protective role in normal tissues and contribute to tumorigenesis when dysregulated, consistent with previous reports^32^.

Differential expression analysis of m6A-modified genes revealed significant associations with several critical biological processes, including cell migration, DNA replication, and transcriptional regulation. Specifically, genes such as *SOX2* and *TOP2A* displayed hypomethylated m6A modifications, with both genes being upregulated in tumor tissues. Therefore, m6A modifications may influence the transcriptional activities of these genes, contributing to tumor progression. These genes are well-established for their roles in regulating cell growth, DNA replication, and immune responses, underlining the importance of m6A modifications in regulating cancer-related processes^33^-^36^.

Our findings also emphasize the heterogeneity of m6A modifications in NSCLC, with a substantial proportion of genes exhibiting differential m6A modification patterns between tumor and normal tissues. Among the 9,644 genes with aberrant m6A modifications in tumors, 5,224 also showed significant changes in RNA expression, reinforcing the role of m6A in regulating protein translation in oncogenic processes^37^. Furthermore, 88.8% of these m6A-associated DEGs were protein-coding, supporting the idea that m6A primarily affects mRNA translation and stability^38^. The Fisher's exact test revealed a strong correlation between m6A modifications and gene expression.

The expression patterns of m6A regulators demonstrated notable heterogeneity across NSCLC tissues, emphasizing the complexity of m6A regulatory mechanisms in tumorigenesis^39^. Key regulators like *METTL3*, *METTL16*, and *CBLL1* (writers), *FTO* and *ALKBH5* (erasers), and *ELAVL1* (readers) exhibited consistent expression patterns across different NSCLC subtypes, suggesting coordinated regulation of m6A modifications. Additionally, 88.8% of m6A regulators, including *ALKBH5*, *METTL3*, *HNRNPA2B1*, *WTAP*, *HNRNPC*, and *FTO*, cooperatively regulated 57.0% of m6A-associated DEGs. This complex network calls for future research into the molecular mechanisms governing m6A regulators in NSCLC and their potential as therapeutic targets^40^-^42^.

Gene expression levels varied significantly across different pathological T stages of NSCLC, indicating the role of m6A modifications in disease progression, particularly metastasis and tumor invasiveness. Several dysregulated m6A-modified genes were associated with patient prognosis. High expression levels of *HMGA1*, *LRFN4*, and *ERO1A* correlated with impaired outcomes, while elevated levels of *SLC2A1* and *VGLL3* were linked to favorable prognosis. These findings suggest that m6A-modified genes could serve as potential prognostic biomarkers for NSCLC, offering insights into personalized treatment strategies based on m6A profiles.

Our study has several important limitations that warrant emphasis. First, the extremely small sample size (five patient pairs) drastically limits statistical power and generalizability, and we have framed this work as a pilot hypothesis-generating study throughout. Second, the absence of orthogonal methodological validation (e.g., m6A-miCLIP or MeRIP-seq) for our nanopore-based m6A calls requires acknowledgment. Third, all prognostic associations are inferred from public TCGA data lacking direct m6A measurement and require validation in independent cohorts with m6A data. While we observed associations between m6A modifications and gene expression, these correlations cannot establish causality without functional validation. Finally, technical limitations in m6A quantification accuracy, particularly for low-abundance transcripts, may introduce bias. Addressing these limitations through expanded cohorts, orthogonal validation, and functional studies will be essential for future research.

In conclusion, our study underscores the significant role of m6A modification in transcriptional regulation by comparing differential m6A and transcriptome profiles between NSCLC and normal tissues. We also identified four m6A-associated DEGs as potential prognostic biomarkers for NSCLC. This research highlights the importance of integrating m6A modification and RNA expression analyses to uncover novel prognostic markers and therapeutic targets. Furthermore, it demonstrates the utility of long-read sequencing technology in studying RNA epigenetic regulation in cancer.

## Supplementary Material

Supplementary figures and tables.

## Figures and Tables

**Figure 2:**
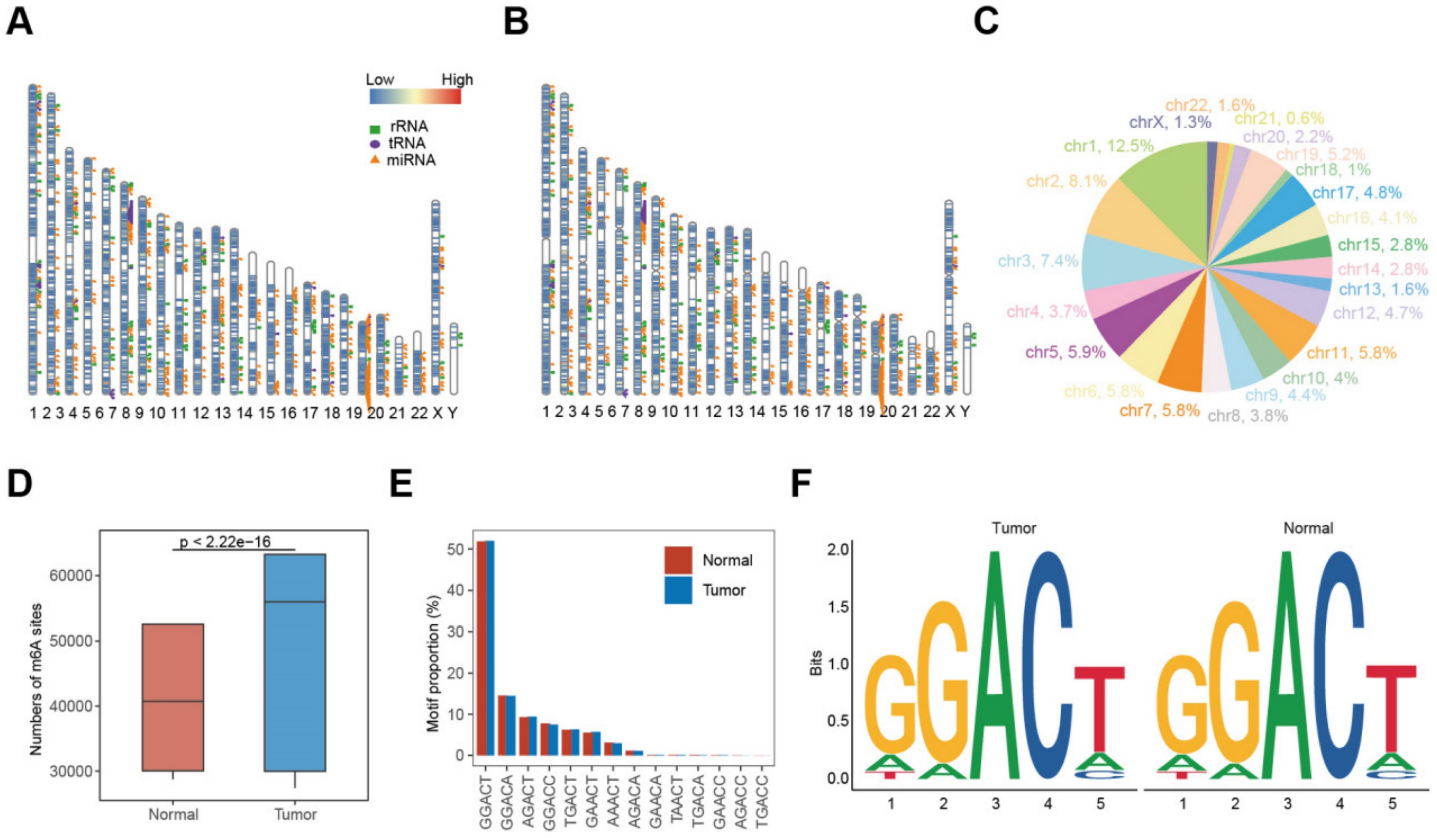
** Experimental design and data reproducibility of m6A profiling in NSCLC. (A-B)** Distribution of m6A sites in tumors** (A)** and normal tissue** (B)** across the genome.** (C)** Proportion of m6A sites on each chromosome. **(D)** Numbers of m6A sites in tumors (blue) and normal tissues (red), with significant differences (Mann-Whitney U test, *P* < 0.0001). **(E)** Proportion of each m6A motif in tumors and adjacent normal tissues. **(F)** Sequence logos showing single-base differences in m6A motifs between tumors (left) and normal (right) tissues.
