# Supplementary figures and images for: Comprehensive Analysis of Aberrant m6A RNA Modifications Identifies Prognostic Biomarkers in Non-Small Cell Lung Cancer
Source: Int J Med Sci. 2025 Oct 24;22(16):4396–405. doi: 10.7150/ijms.119651 (PMC12595339; doi:10.7150/ijms.119651)

Supplementary Figures


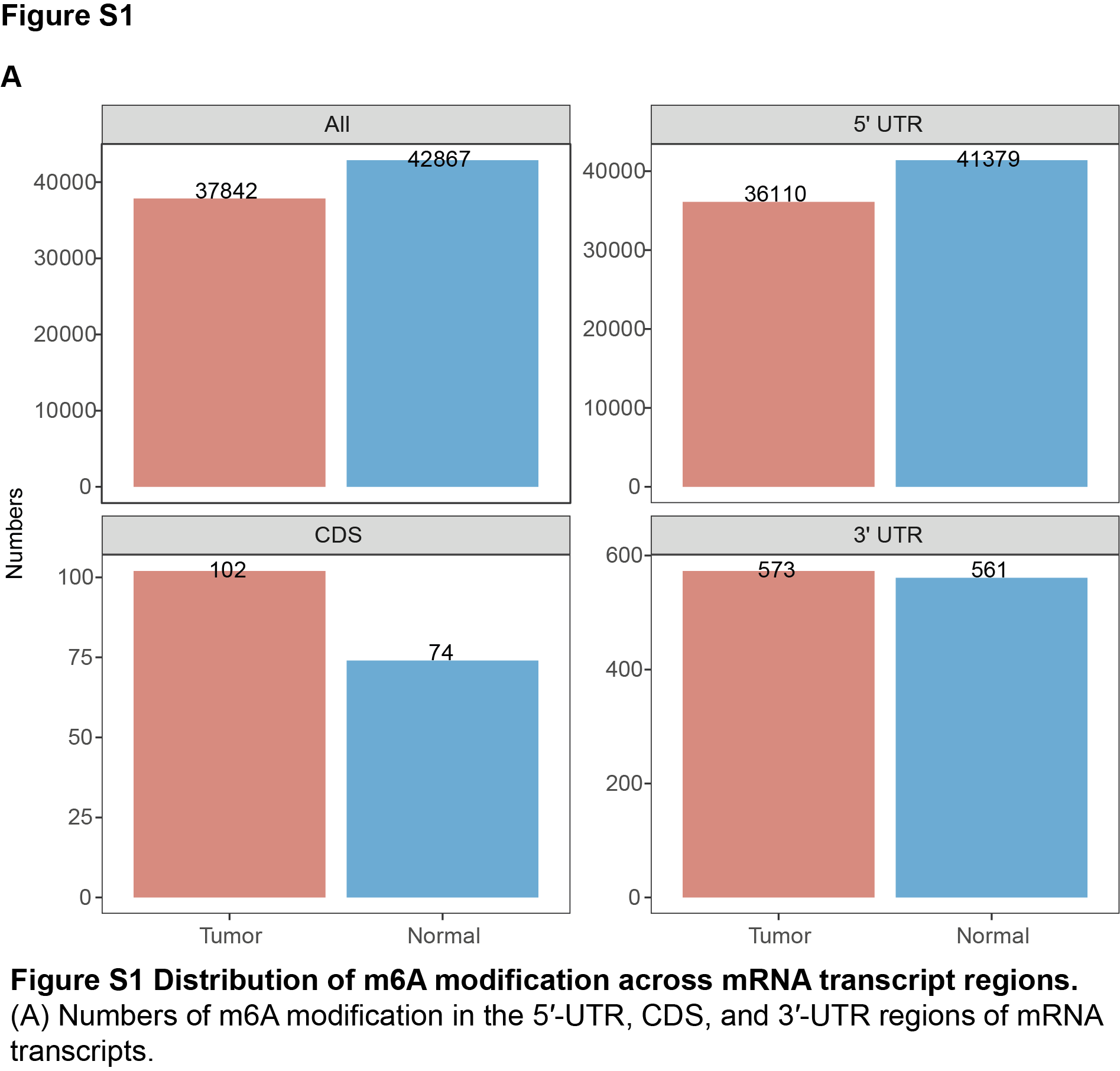


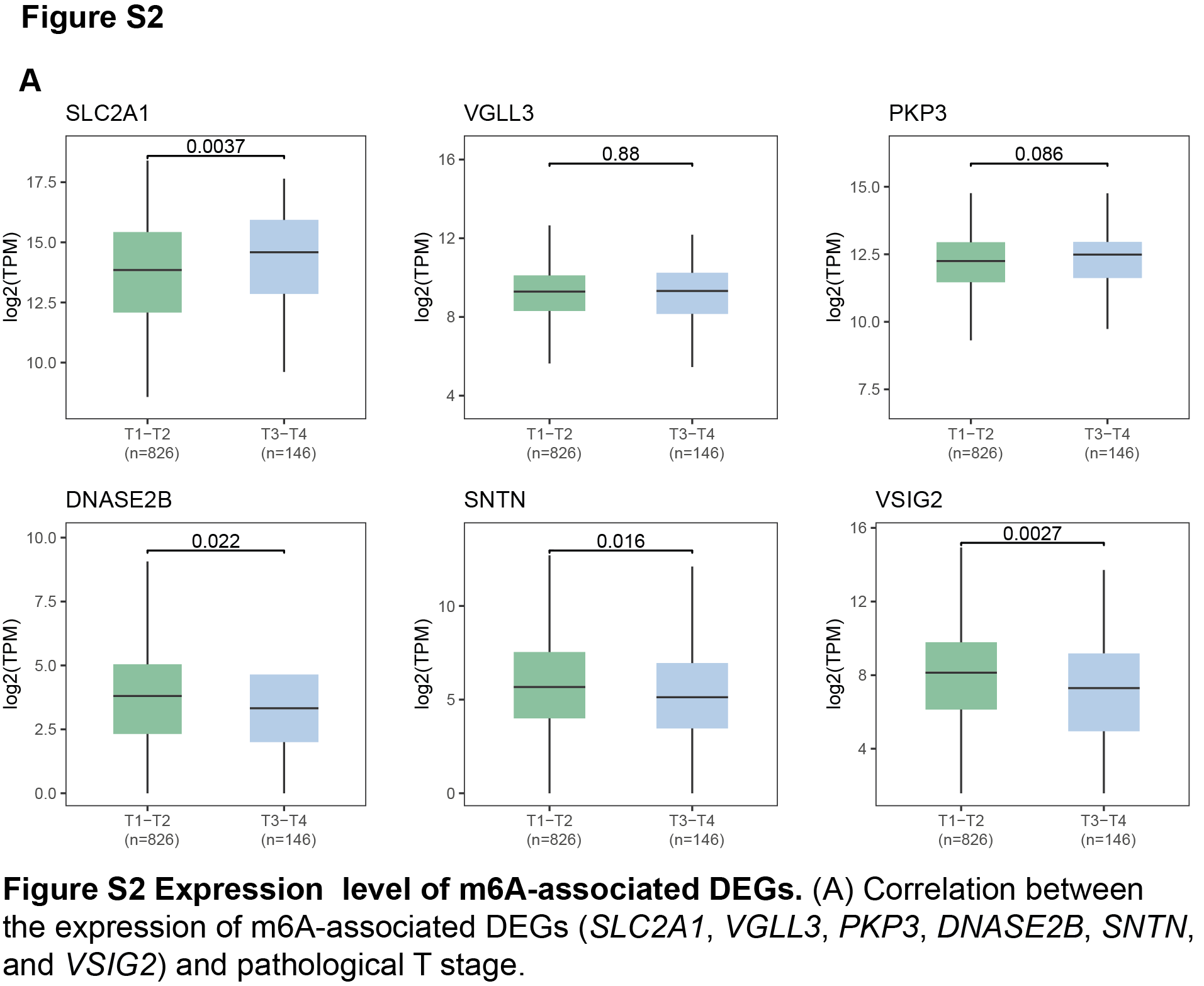


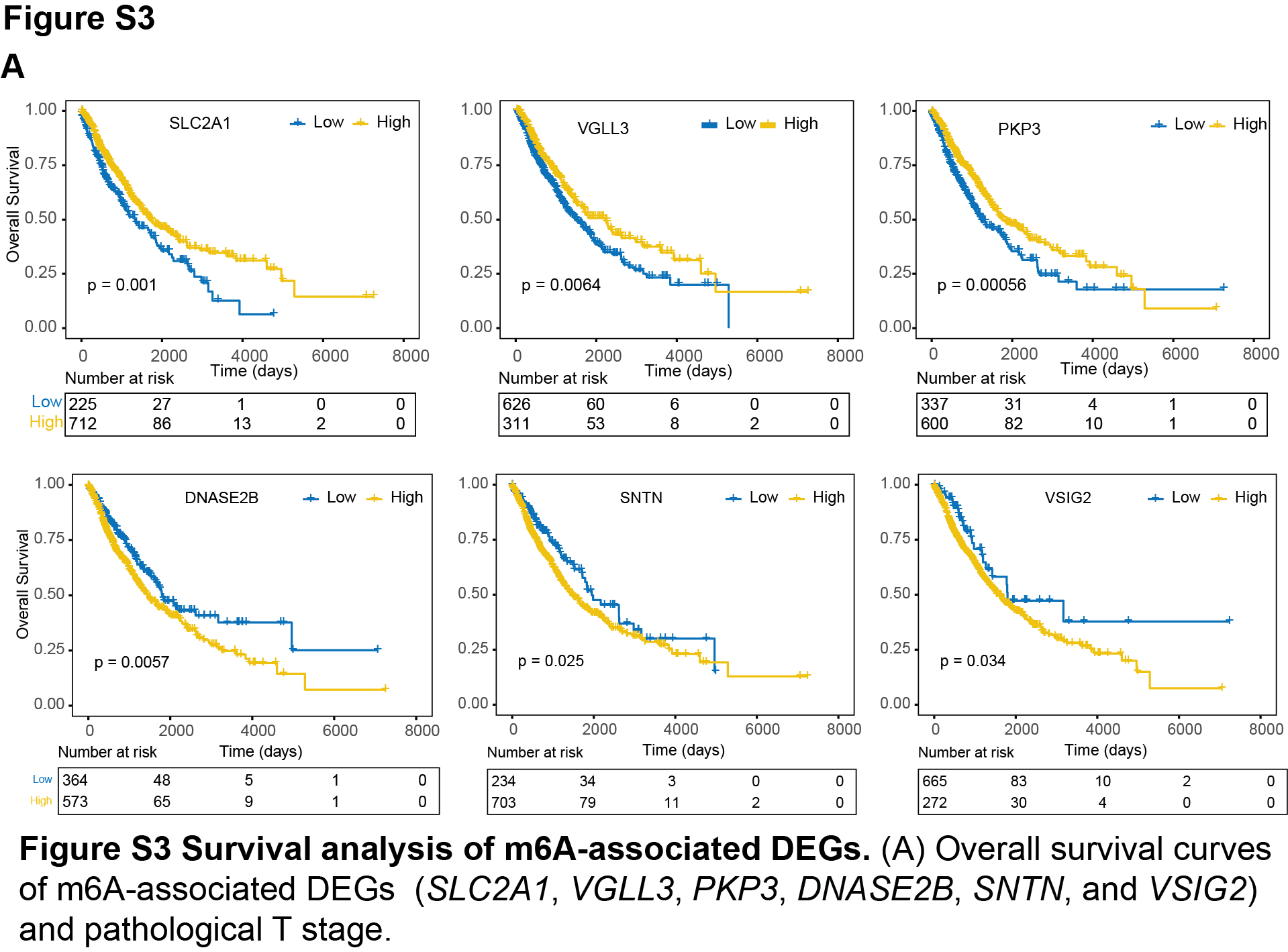

Supplement: Supplementary file 1 — Supplementary figures and tables. [file ijmsv22p4396s1.zip › Supplementary material/Supplementary Figures.docx]
